# Future Cognitive Ability: US IQ Prediction until 2060 Based on NAEP

**DOI:** 10.1371/journal.pone.0138412

**Published:** 2015-10-13

**Authors:** Heiner Rindermann, Stefan Pichelmann

**Affiliations:** Department of Psychology, Technische Universität Chemnitz, Chemnitz, Germany; Nathan Kline Institute and New York University School of Medicine, UNITED STATES

## Abstract

The US National Assessment of Educational Progress (NAEP) measures cognitive competences in reading and mathematics of US students (last 2012 survey *N* = 50,000). The long-term development based on results from 1971 to 2012 allows a prediction of future cognitive trends. For predicting US averages also demographic trends have to be considered. The largest groups’ (White) average of 1978/80 was set at *M* = 100 and *SD* = 15 and was used as a benchmark. Based on two past NAEP development periods for 17-year-old students, 1978/80 to 2012 (more optimistic) and 1992 to 2012 (more pessimistic), and demographic projections from the US Census Bureau, cognitive trends until 2060 for the entire age cohort and ethnic groups were estimated. Estimated population averages for 2060 are 103 (optimistic) or 102 (pessimistic). The average rise per decade is *dec* = 0.76 or 0.45 IQ points. White-Black and White-Hispanic gaps are declining by half, Asian-White gaps treble. The catch-up of minorities (their faster ability growth) contributes around 2 IQ to the general rise of 3 IQ; however, their larger demographic increase reduces the general rise at about the similar amount (-1.4 IQ). Because minorities with faster ability growth also rise in their population proportion the interactive term is positive (around 1 IQ). Consequences for economic and societal development are discussed.

## Introduction

Since the seminal overview of James Flynn in 1984 “IQ of Americans: Massive gains 1932 to 1978” [1] it is well known to a broader scientific community that cognitive test results in the US and other modern countries are rising from generation to generation. The first time it was discovered by Rundquist [2]. Later this trend was described by Lynn for Japan [3], Flynn for the US [1] and many other researchers. According to meta-analyses of Pietschnig and Voracek [4] and Trahan et al. [5] across different decades and countries, the average effects per decade were around *dec* = 2 to 3 IQ points with, in the last decades, some indications of larger improvements in lower scoring groups and countries who are catching up [6–8]. Also, according to NAEP (National Assessment of Educational Progress), the lower scoring groups within the US (African Americans and Hispanics) showed a faster rise leading to reduced ability gaps [9].

Future challenges are expected to raise cognitive demands: Cognitive tasks at the workplace as well as in daily life and in organization, maintenance and especially innovation are rising, e.g. management of an airport, supply and sale in industry, information logistics in administration or improvements in technology and software. Researchers and economists fear that the US and other countries are not prepared for the expected larger demand for non-routine analytical-cognitive jobs causing an obstacle for future economic growth [10–12]. Cognitive ability is crucial for being successful in complex job tasks [13–16]. Thus any positive development of ability as in the past should have a positive impact on functionality, productivity, and political-cultural development of a society.

Considering the positive past trend, a negative outlook by many authors is at first sight rather astonishing. However, they refer to internationally comparing studies as TIMSS and PISA in which the US students achieved only mediocre to third-class results. For instance, in PISA 2009 mathematics only 32% of US students reached the proficiency level compared to Australia with 44%, Canada with 50% and Singapore with 63% [17]. Similarly, Lawrence Summers complained that “American students lag badly and pervasively.” [18] High school graduation rates peaked in the early 1970s [19]. Because cognitive abilities are crucial for economic growth, Hanushek et al. see the future prosperity under threat (“Endangering prosperity”). They estimated, if the US ability level improved to the Canadian level, their GDP per capita would be around $70,000 US higher at the end of the 21^st^ century [17]. A further critical voice is Vivek Wadhwa who sees the US technological and economic future quite skeptical because highly skilled immigrants are discouraged by restrictive immigration laws [20]. Finally, Educational Testing Service researchers fear due to demographic changes a decline in average cognitive test results with negative consequences for wealth: “Unless we are willing to make substantial changes, the next generation of Americans, on average, will be less literate and have a harder time sustaining existing standards of living.” [21]

More generally, researchers have observed a decline in basic cognitive ability and mental speed in Western countries [22]. Additionally, immigration of people from countries with, on average, lower educational and ability level, as indicated by international statistics (e.g. Human Development Report), student assessment studies (e.g. PISA) and psychometric intelligence test studies (e.g. using the figural Raven Matrices) may create a serious obstacle for future competence development, at least for the continuation of the “FLynn effect”. The secular rise of test results and abilities may be turned into the reverse [23]. The ability levels of important and large immigrant groups are below average (e.g. in Europe from Middle East and African countries) [24–26] but the increase of their ability levels is also noticeable, e.g. immigrants from Turkey in The Netherlands achieved a rise from IQ 81 to 88 within one generation [27]. E.g., in PISA 2009 [25], in Finland natives achieved in reading 538 student assessment scale points (SAS, international developed countries’ mean *M* = 500, standard deviation *SD* = 100), but second generation immigrants *SAS* = 493 and first generation immigrants only *SAS* = 449, a difference of 89 SAS-points equivalent to around two to three school years of progress.

Regarding immigration effects, there are large differences between countries [26]: For example, for the US there is gap of 4 IQ points between natives and immigrants favoring natives. Considering the shares of both groups, this leads to an immigration based ability loss of 1 IQ. For Canada the gap is only 1 IQ (-0.30 IQ loss). For Australia the gap is even reversed with –1 IQ (immigrants higher) leading to an immigration based gain of +0.30 IQ. The largest gains are given for rich Arab states of the Persian Gulf (up to +7 IQ in the Emirates). Larger losses are observable for Central and Western Europe (around –1 to –2 IQ).

For understanding US cognitive ability development, especially important are gaps between racial-ethnic groups such as Whites, Blacks, Hispanics, and Asians. Whites were and are the largest group with most impact on politics, institutions and economics in the US [28]. Their proportion among the population is declining and their ability rise (FLynn effect), according to NAEP, is quite flat until 2012 (see Table 1). Hispanics form now the second largest group, their population share is continuously rising, and their ability gap towards Whites is declining in the last two generations (Table 1). Politically most important for the US is the ability gap between Whites and Blacks (see the past discussions on causes and consequences [29]). In NAEP until 2008, it decreased from around 15 IQ points to 10 IQ points [9] (see also Table 1). However, both groups’ rises lost speed in the last two decades. The NAEP results are corroborated by other studies. The White-Black-gap-narrowing nearly stopped in the late eighties (highest educational degrees, Graduate Record Exam, SAT, ACT) [29–31].

**Table 1:** Calculated NAEP based cognitive ability scores in IQ from 1978/80 to 2012.

	White	Black	Hispanic	Asian	Average
1978/80	100.00	81.46	86.92	100.89	97.27
1982/84	99.99	86.31	88.34	99.98	97.47
1986/88	100.75	90.03	90.35	101.73	98.65
1990	101.60	90.94	91.25	100.70	99.13
1992	102.32	88.97	92.56	102.67	99.63
1994	102.07	90.01	90.63	101.60	99.24
1996	102.19	90.20	91.36	100.11	99.37
1999	102.43	89.06	92.57	102.87	99.59
2004	101.69	89.56	90.53	102.06	98.62
2008	102.29	90.45	92.30	103.92	98.68
2012	102.28	91.14	93.60	105.05	98.88

NAEP: National Assessment of Educational Progress. Due to alternating surveys, the survey 1978 (mathematics) and 1980 (reading) combined, 1982 (mathematics) and 1984 (reading) combined, 1986 (mathematics) and 1988 (reading) combined. Benchmark: White 1978/80 average in reading and mathematics. Based on the oldest age-group, the 17-year-old students. Average calculated based on ethnic proportions of survey participants.

What development for the US, for certain groups, and the gaps between them can be predicted for the future? We try to answer this question by taking past development as basis for predicting future trends and combining these trends with estimates of US population development. The aims of this paper are:

1. First, newer, recently published data on NAEP until 2012 allow analyzing if the trends of the past decades continue, the ability rise at a lower pace, and a reinstatement of the gap shrinking, which was flat in the last 20 years. Additionally, using other sources of NAEP data and more sophisticated analysis methods enable an examination of the first trend analysis [9].
2. Second, we try to predict future cognitive development for the US. For this purpose, we draw on past ability trends as measured by NAEP using different periods, minimum and maximum trends and add additional information on population development.
3. According to cognitive human capital theory, cognitive trend predictions are used for estimating economic effects.

We use NAEP data based on two time periods, first from a longer period from 1978/80 to 2012 with an, on average, larger ability increase leading to a more optimistic prediction and second from a more recent period from 1992 to 2012 with a flatter development leading to a more pessimistic prediction. We add information on population development and we try to calculate lower and upper bounds of the possible development. Without having further knowledge on determinants of historic development of ability levels and their change a prediction based on the past development in different periods and additional information on demographic trends is the best possible method.

We use the 17-years-old student group. They will form the later workforce and are the best predictor for the ability level of younger and older adults shaping the society. Earlier ability levels may peter out or represent only a faster cognitive development in young age. At the level of nations, student assessment approaches (as PISA and TIMSS) correlate with adult assessments (Programme for the International Assessment of Adult Competencies 2012, PIAAC) at *r* = .67 in a common 23 country sample (corrected for range restriction the correlation is *r* = .95). Thus, at the national level measurement in student age can be taken as an approximation of the ability level of adults.

Based on these estimations and taking formula from cognitive human capital theory we predict economic effects. They can be compared to estimations based on other data and formula. Finally, we discuss less predictable political effects, e.g. of ability heterogeneity or growing homogeneity.

## Method

### 1.1. Data sources

*Long-Term Trend NAEP*: Our study is based upon data measured by the Long-Term Trend Assessment of the National Assessment of Educational Progress (NAEP). NAEP is intended to measure US students’ cognitive achievement in reading and mathematics to give politicians, school administrators, teachers and scientists a global feedback on a generation’s achievement level, the quality of education and their development across age and time. NAEP provides representative statistics on academic abilities of 9-, 13- and 17-year-old US students in reading and mathematics. For both, reading and mathematics, their first assessments date back to the early 1970s (reading 1971, mathematics 1973). Since then, these assessments have been regularly repeated at intervals of two to five years. The most current assessments were taken in 2012.

According to the National Center for Education Statistics, reading assessment questions of NAEP focus on “locating specific information, identifying main ideas, and making inferences across a passage to provide an explanation” [32] whereas mathematics assessment questions lay attention on “knowledge of mathematical facts, their understanding of basic measurement formulas as applied in geometric settings, their ability to carry out computations … and applying mathematics to daily living skills” [33].

There are similarities between scholastic assessment and psychometric IQ tests in *item content* (both usually contain verbal and numerical items), *cognitive demands* and *processes* in solving tasks (e.g. analysis of relations, finding of rules and applying them, categorization, forming of concepts, retrieval of knowledge), *applied cognitive abilities* (speed, concentration, working memory, fluid intelligence, knowledge), *influence of test familiarity* and in *validity* [33]. Empirically, results of scholastic tests and psychometric IQ are highly correlated. Kaufman, Reynolds, Liu, Kaufman, and McGrew reported a latent correlation of *r*_l_ = .83 (*N*>2,000) between the Woodcock-Johnson Tests of Cognitive Abilities and the Kaufman Test of Educational Achievement [34]. This result is backed by studies of real-world school achievement: e.g. *r*_l_ = .81 between General Certificate of Secondary Education scores and Cognitive Abilities Test (*N*>70,000) [35] or *r*_l_ = .86 between Scholastic Assessment Test and Armed Services Vocational Aptitude Battery (*N*>900) [36]. At the national level (the one we will use in our analysis) the correlations are even higher (e.g. *r* = .89, *N* = 99 countries) [37].

Aside from similarity in items, demands, processes and useful abilities, the *common causes* of individual development and individual and group differences in both constructs contribute to the observed high correlations (e.g. factor mental speed [38]). A further reason is *within person developmental interaction of abilities*: Fluid intelligence (thinking, in a narrower sense intelligence) and crystallized intelligence (knowledge) co-develop in mutual stimulation. All determinants relevant for intelligence development such as genes, health, education in family and school, class mate ability etc. are also relevant as determinants for achievement in schools and for results in both kinds of tests. For all these empirical and theoretical reasons, we can use NAEP test results measuring reading and mathematics as good indicators of what is usually called intelligence or cognitive ability. Hence, it is possible to convert NAEP scores to IQ scores and vice versa. Due to its link to school curricula and knowledge (especially in mathematics), the NAEP test is more inclined to crystallized than fluid intelligence.

Since 1978/80 (mathematics/reading) students taking part in the NAEP measurements are subdivided in six categories based on their ethnicity: (1) White (not Hispanic), (2) Black (not Hispanic), (3) Hispanic, (4) Asian American or Pacific Islander, (5) American Indian or Alaska Native, and (6) Unclassified. For each of these categories detailed statistics like average score, standard deviation, and group size are provided online by the National Center for Education Statistics [39]. The group sizes of American Indian or Alaska Native and Unclassified fluctuated between zero and two percent since 1978/80. Hence, both were excluded from our calculations due to insufficient data.

### 1.2. 2012 National Population Projections

To predict the overall development of US citizens’ cognitive ability, reliable statistics on population growth for the different ethnic groups are necessary. In 2012 the United States Census Bureau (USCB) published national population projections for the years 2015 to 2060 [40]. These projections are based on 2011 USCB population estimates combined with assumptions on fertility, mortality and net migration. A detailed report on the applied methodology is available through USCB’s website. There are four projections series available: The main series as well as three alternative series based on different estimates for future net immigration. Each series includes population projections for every five years starting with 2015. We chose the main series for our calculations. Since the population projection ends 2060, we used the same time period.

As we wanted to make the NAEP data of 2012 our reference point, we utilized population estimations of the USCB Population Division for the year 2012 [41] as well. American Indians and Alaska natives as well as native Hawaiian and other Pacific islanders were excluded, as we had no sufficient corresponding NAEP data.

Because NAEP uses student population data but USCB estimates group shares for the total population there are differences–student population anticipates later total population development. E.g. for 2012 there is a 7%-difference for Whites (NAEP: 56% vs. USCB: 63%). This results in a minor difference in the total population average IQ of 0.62 IQ points.

### 1.3. Cognitive ability projections based on population size of groups

Our methodology to predict US citizens’ future cognitive abilities follows Rindermann and Thompson’s past development analysis [9]. First, we converted NAEP scores to IQ scores separately for mathematics and reading. IQ scores are normally distributed with a mean score of *M* = 100 and a standard deviation of *SD* = 15. Such a definition does not exist for the NAEP scale which ranges between 0 and 500. Instead the general scale *M* and *SD* values have to be estimated based on average scores and *SD’*s published for the different age groups, ethnicities, and years.

Different to Rindermann and Thompson [9] we did not use the first reading (1971) and mathematics measurement as benchmark for the 100 and 15 standard but the surveys 1978 (mathematics) and 1980 (reading). Reasons: 1. These were the first survey years NAEP gave results based on six race-ethnicity groups. Because these groups have noticeable different ability levels and trends and their proportions develop differently, a prediction has to consider group affiliation. 2. Additionally, international cognitive ability comparisons are anchored in the year 1979 [42]. The same benchmark (1978 and 1980 vs. 1979) enables trend comparisons.

Similar to Rindermann and Thompson [9], we used the largest group’s (White) achievement average and *SD* as benchmark (as mentioned from the survey of 1978/80, mathematics/reading). The white students represented the vast majority of students in those years and they had been formative for the US. This also makes American means and developments more comparable to those in European countries.

Finally, we concentrated our analysis on the 17 year old (not primary 9-year- or secondary 13-year-old students). Their ability level should be the best indicator of the whole population’s ability level and the best estimation of the ability level of the coming workforce.

For each race-ethnicity and subject, NAEP scores were converted to IQ scores using the following formula:

$${\bar{IQ}}_{s,y,r}=\frac{{\bar{NAEP}}_{s,y,r}-{\bar{NAEP}}_{s,78/80,white}}{{SD}_{s,78/80,white}}*\;15+100$$

The indices are abbreviations for subject: mathematics or reading (*s*), year of NAEP survey (*y*), specific race and ethnic group (*r*), and data of white students in 1978/80 (*78/80*, *White*).

To predict the average increase in IQ points for every race-ethnicity and subject, we calculated regressions based on the calculated IQ scores. To account for possible changes in average IQ increase over time, linear regressions were based on IQ scores of two different time periods (*tp*): 1978/80 to 2012 (last 34 years/32years) as well as 1992 to 2012 (last 20 years). The first time period was chosen, as it included all available data, while the latter one represents the changes during the more recent historical period covering effects of technological innovation relevant for learning (e.g., home computers, notebooks and tablets, Internet, smart phones). Furthermore 20 years (the 1992–2012 period) roughly represent the age of the 17-year-olds and the IQ changes during their lifetime. We included both time periods, as it allows for comparison and judgment of the trends’ stability (or linearity) by readers. Shorter intervals lead to more unstable predictions. We did not apply linear regression as we did not want to have an unspecific intercept. Instead we used the data of 2012 as our intercept, whereas *m* is the slope estimated based on the regression:

$${\bar{IQ}}_{s,y,tp,r}={\bar{IQ}}_{s,2012,r}+m_{s,tp,r}\;*\;(y-2012)$$

In the longer interval a larger ability rise was observable leading to more optimistic predictions than the one based on the shorter interval (more pessimistic predictions). Afterwards, we calculated the mean regression function of both subjects (reading, mathematics). Hence the IQ in a specific year of a specific group, based on one of the two time periods, can be described by the following formula:

$${\bar{IQ}}_{y,tp,r}={\bar{IQ}}_{2012,r}+m_{tp,r}\;*\;(y-2012)$$

To project the average IQ of US citizens in a specific year for one of our two time periods, the group-dependent IQ scores were put in relation to the 2012 National Population Projections’ estimated population proportion for each race-ethnic group (p_r_).

$${\bar{IQ}}_{y,tp}=\sum_{r}p_{y,r}\;*\;{\bar{IQ}}_{y,tp,r}$$

We generated two other models for each time period based on these calculations. We selected the *maximum* (*m*_*max*,*tp*_, actually Asians, being more “optimistic”) and minimum (*m*_*min*,*tp*_, actually Whites, being more “pessimistic”) slope of all four race-ethnic groups and used them for all four groups:

$${\bar{IQ}}_{\text{max},y,tp,r}={\bar{IQ}}_{2012,r}+m_{\text{max},tp}\;*\;(y-2012)$$

$${\bar{IQ}}_{mín,y,tp,r}={\bar{IQ}}_{2012,r}+m_{\text{min},tp}\;*\;(y-2012)$$

These models were combined with the estimated population proportion for each race-ethic group as well:

$${\bar{IQ}}_{\text{max},y,tp}=\sum_{r}p_{y,r}\;*\;{\bar{IQ}}_{\text{max},y,tp,r}$$

$${\bar{IQ}}_{\text{min},y,tp}=\sum_{r}p_{y,r}\;*\;{\bar{IQ}}_{\text{min},y,tp,r}$$

We generated these models as worst and best case scenarios (what is socially possible) for each corresponding time period. One reviewer suggested checking whether the ethnic differences change with age to estimate their differences for adults, the age of the workforce. We only found minor trends for 2012, but unstable patterns for the other measurement points. Therefore we did not change the prediction method.

### 1.4. Effects

Four effect sizes were calculated to analyze the projected IQ scores in more detail:

(1) The effect as if all groups had the same IQ slope (e_base_) with the slope of the white students as baseline.

$$e_{base;y,tp}=(y-2012)\;*\;m_{tp,white}$$

(2) The additional effect due to the higher slopes of the minorities (e_minor_).

$$e_{\text{min}\;or;y,tp}=(y-2012)\;*\;\sum_{r}p_{y,r}\;*\;(m_{tp,r}-m_{tp,white})$$

(3) The effect of the population change (e_pop_).

$$e_{pop;y,tp}=\sum_{r}{\bar{IQ}}_{2012,tp,r}\;*\;(p_{y,r}-p_{2012,r})$$

(4) The interaction of population change and the higher slopes of the minorities (e_p&m_).

$$e_{p\&m;y,tp}=(y-2012)\;*\;\sum_{r}\left(m_{tp,r}-m_{tp,white})\;*\;(p_{y,r}-p_{2012,r}\right)$$

The year of the latest NAEP assessment (2012) was chosen as a reference point. Please see Appendix for the derivation of the equations.

### 1.5. Economic effects

To estimate economic effects we used the same procedure as in Rindermann and Thompson [9]: As currency unit was taken the 2010 per capita GDP based on 2010/2011 dollar purchasing-power-parity (World Economic Outlook Database April 2011). The correlation between cognitive ability and wealth is estimated at *r* = .53. In regressions one IQ-point corresponds to a gain of $810 US Dollar per capita per year and we applied it to IQ-transformed NAEP-changes between 2012 and 2060 without considering inflation or general economic growth during this time.

## Results

### 2.1. Calculated IQ Scores of 1978/80 to 2012

The calculated IQ scores for the past trend are presented in Table 1. The year of assessment for mathematics and reading differed between 1978 and 1980 by a two years gap. We combined the calculated IQ scores of 1978 and 1980, 1982 and 1984, as well as 1986 and 1988. The results are based on the oldest NAEP student group, the 17-year-olds.

The cognitive ability level in reading and mathematics relative to the benchmark of Whites in 1978/80 rose for all groups: For Whites from 100.00 to 102.28, for Blacks from 81.46 to 91.14, for Hispanics from 86.92 to 93.60 and for Asians from 100.89 to 105.05 in 2012. The terms “Whites”, “Blacks” etc. refer to the categories by NAEP, corresponding to students in the US with European, sub-Saharan African background etc. The total average is not equal to the simple mean. Instead, the average is weighted by the proportion of students of each ethnic group in the corresponding year (excluding others or not categorized students, max. 2.5%). Thus, the mean group increase, arithmetically averaged from 1978/80 to 2012 with +5.70 IQ, is larger than the empirically observed average of +1.61 IQ. As the proportions of groups with lower ability levels increased more (especially Hispanics) and the proportions of groups with stronger rise are smaller (Blacks and Asians), the total rise is below the arithmetic average rise.

From 1992 to 2012 Whites did not achieve an ability rise but all three minority groups did. This led to a restart of the gap shrinking between Whites and Blacks and Whites and Hispanics, but it also increased the gap between Asians and all other groups. The Asian strong rise could be caused by better language proficiency or better education within a US-American Asian group. However, it could also be due to an ethnic change within the heterogeneous Asian group, e.g. from Filipinos to Chinese. (Information on ethnic background of Asian immigrants and its change make such an interpretation not plausible [43])

The slopes of the regressions for both time periods are listed in Table 2. In the longer period (1978/80 to 2012), on average, Asian students showed the largest IQ increase with 1.67 IQ points per decade. Taking only the last 20 years into consideration (1992 to 2012), it is again the Asian students with 2.00 IQ points per decade who show the highest increase. Predictions for Asian students (a rather small group) based on the longer period (1978/80 to 2012) result in more optimistic results, whereas predictions on the more recent period (1992 to 2012) are more pessimistic.

**Table 2:** Regression based IQ slopes from 1978/80 to 2012 and 1992 to 2012.

	Year	White	Black	Hispanic	Asian
Mathematics	1978/80-2012	0.0914	0.1636	0.1990	0.1186
	1992–2012	0.0211	0.0719	0.0829	0.1166
Reading	1978/80-2012	0.0016	0.1243	0.1089	0.2150
	1992–2012	-0.0098	0.1255	0.1659	0.2824
Average	1978/80-2012	0.0465	0.1439	0.1539	0.1668
	1992–2012	0.0056	0.0987	0.1244	0.1995

Slope: unstandardized m (unit: IQ increase per year). Because of small values four digits are presented. In the longer period from 1978/80 to 2012 the rises are slightly larger (arithmetic mean across the four groups: *m* = 0.13) than in the shorter and more recent period from 1992 to 2012 (arithmetic mean: *m* = 0.11).

Compared to Rindermann and Thompson (Table 2) [9] there are only minor deviations (Table 2): Whites *dec* = 0.45 vs. 0.46, Blacks *dec* = 2.35 vs. 1.44, Hispanics *dec* = 1.55 vs. 1.54. For the entire NAEP group (difference between 1978/80 and 2012) the average decadel effect is *dec* = 0.50 vs. 0.30. The remaining differences are mainly due to differing intervals (1971–2008 vs. 1978/80-2012).

### 2.2. Projected IQ Scores of 2012 to 2060

Table 3 shows the percentages of participants of the NAEP for each race-ethnicity. Sometimes, the total percentages exceed 100% due to rounded values in the NAEP data. The population projections of the US Census Bureau for these groups are presented in Table 4. The percentage of the total population for these four groups is slowly decreasing (2012: 97.19%, 2060: 94.26%). Hence, the projection error due to ignoring small ethnic groups like Native Americans might slightly increase for later years.

**Table 3:** Percentage of race-ethnic groups in NAEP (students).

	Year	White	Black	Hispanic	Asian	Sum
Mathematics	1978	83	12	4	1	100
	1982	81	13	5	2	101
	1986	78	14	5	2	99
	1990	73	16	7	3	99
	1992	75	15	7	3	100
	1994	72	15	9	2	98
	1996	71	15	9	3	98
	1999	71	15	10	4	100
	2004	68	12	14	4	98
	2008	59	14	19	5	97
	2012	56	13	22	6	97
Reading	1980	83	12	4	1	100
	1984	77	14	7	2	100
	1988	77	15	6	2	100
	1990	74	16	7	3	100
	1992	75	15	8	3	101
	1994	72	15	8	3	98
	1996	72	15	9	3	99
	1999	72	14	9	4	99
	2004	67	12	15	4	98
	2008	59	15	18	5	97
	2012	56	14	22	6	98

Percentages above 100 are due to imprecision of the already rounded NAEP data.

**Table 4:** Percentage of race-ethnic groups in US Census Bureau population estimations.

	White	Black	Hispanic	Asian	Sum
2012	62.98	12.34	16.89	4.98	97.19
2015	61.75	12.40	17.76	5.12	97.03
2020	59.69	12.51	19.10	5.46	96.77
2025	57.61	12.60	20.49	5.81	96.51
2030	55.46	12.68	21.94	6.15	96.23
2035	53.26	12.75	23.44	6.49	95.94
2040	51.02	12.83	24.97	6.81	95.63
2045	48.78	12.92	26.48	7.12	95.30
2050	46.61	13.00	27.95	7.40	94.96
2055	44.53	13.08	29.34	7.65	94.61
2060	42.58	13.16	30.64	7.88	94.26

Table 5 shows the projected IQ scores from 2012 to 2060 for each group as well as the whole population based on 1978/80 to 2012 NAEP scores and the National Population Projections. Table 6 shows the projected IQ scores based on the NAEP scores of 1992 to 2012. The average rise per decade is *dec* = 0.76 or 0.45 IQ points (optimistic vs. pessimistic model).

**Table 5:** Projected IQ scores for 2012 to 2060 based on 1978/80 to 2012 NAEP scores (optimistic).

	White	Black	Hispanic	Asian	Population
2012	102.28	91.14	93.60	105.05	99.50
2015	102.42	91.57	94.06	105.55	99.66
2020	102.65	92.29	94.83	106.38	99.98
2025	102.88	93.01	95.60	107.21	100.31
2030	103.11	93.73	96.37	108.05	100.65
2035	103.34	94.45	97.14	108.88	101.02
2040	103.58	95.17	97.91	109.72	101.41
2045	103.81	95.89	98.68	110.55	101.81
2050	104.04	96.61	99.45	111.38	102.24
2055	104.27	97.33	100.22	112.22	102.70
2060	104.51	98.05	100.99	113.05	103.17

Projection based on the longer interval 1978/80 to 2012 with stronger rise leading to a rather optimistic prediction. Benchmark: Reading and mathematics average of white 17-year-old students 1978/80. 2012 also predicted.

**Table 6:** Projected IQ scores for 2012 to 2060 based on 1992 to 2012 NAEP scores (pessimistic).

	White	Black	Hispanic	Asian	Population
2012	102.28	91.14	93.60	105.05	99.50
2015	102.29	91.44	93.97	105.64	99.56
2020	102.32	91.93	94.59	106.64	99.70
2025	102.35	92.42	95.21	107.64	99.86
2030	102.38	92.92	95.84	108.64	100.04
2035	102.41	93.41	96.46	109.63	100.25
2040	102.43	93.90	97.08	110.63	100.48
2045	102.46	94.40	97.70	111.63	100.73
2050	102.49	94.89	98.32	112.63	101.01
2055	102.52	95.38	98.95	113.62	101.32
2060	102.55	95.88	99.57	114.62	101.66

Projection based on the longer interval 1992 to 2012 with weaker rise leading to a rather pessimistic prediction. Benchmark: Reading and mathematics average of white 17-year-old students 1978/80. 2012 also predicted.

**Fig 1:**
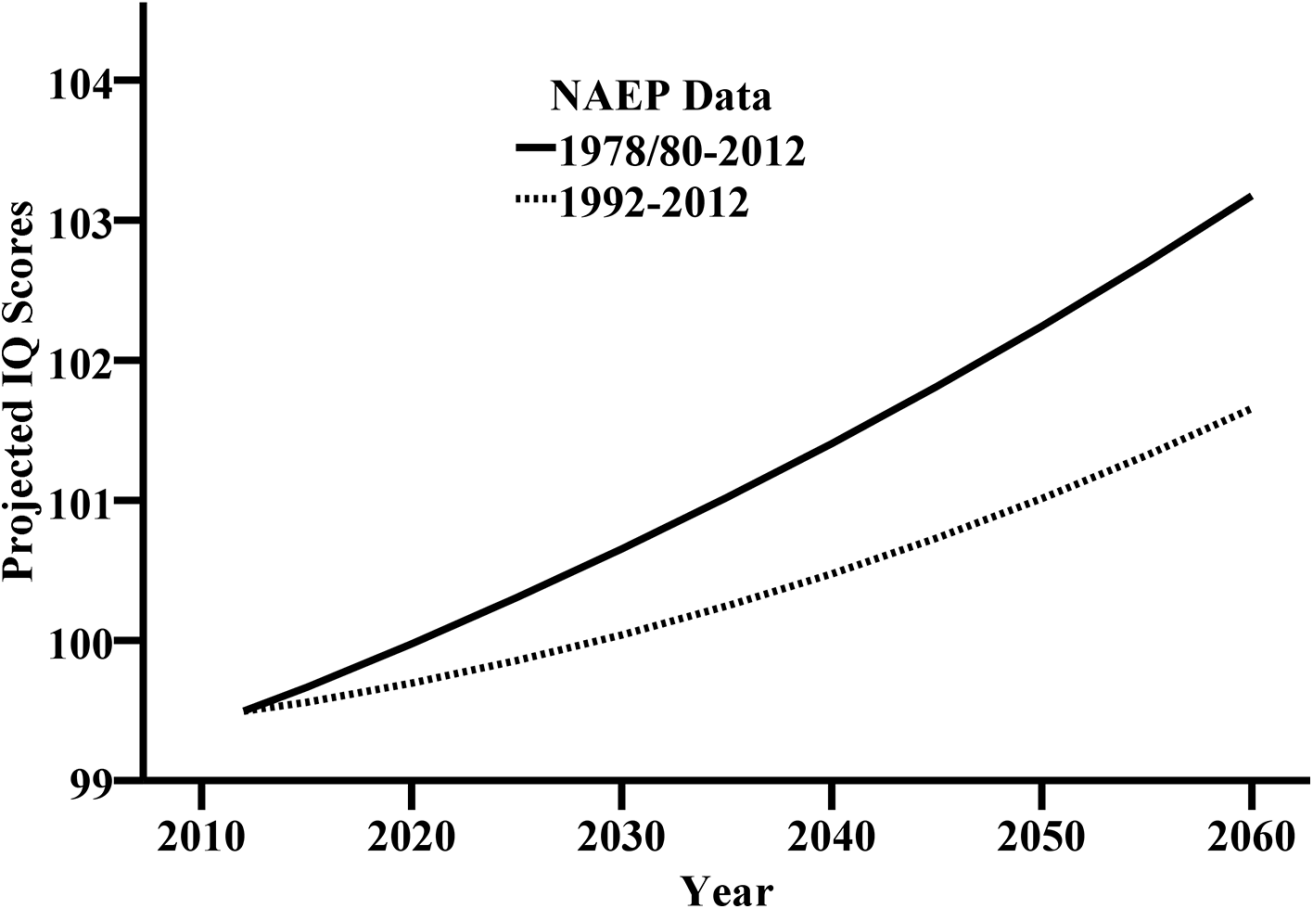
Projected IQ development for US students and young adults based on a longer or shorter NAEP interval.

White-Black gaps from currently 11.1 IQ decrease to 6.5 IQ (optimistic model, Table 5) or 6.7 IQ (pessimistic model, Table 6), White-Hispanic gaps from 8.7 IQ to 3.5 IQ (op.) or 3.0 IQ (pe.), and Asian-White gaps increase from currently 2.8 IQ to 8.5 IQ (op.) or 12.1 IQ (pe.) resulting in a distinctive top Asian group at around 114 IQ. It seems noteworthy that the projected White-Black and White-Hispanic gaps are approximately the same for both models. As mentioned before, the IQ predictions for the Asian students are higher in the pessimistic model. Hence, the projected White-Asian gaps yield the biggest difference when comparing both models. The predictions of both models are combined in Fig 1. The run of the curve is not linear. The used regressions for the prediction are linear but the population changes are curvilinear.

### 2.3. Calculated Effect Sizes

As we used the IQ scores of 2012 as a reference point for our effect size calculations, Table 7 (based on past 1978/80 to 2012 NAEP trend) and Table 8 (based on 1992 to 2012 NAEP trend) show the effect sizes of 2015 to 2060 in five-year intervals, as well as the average effect per year for each of our four postulated effects and the total effect (e_total_). Using the longer past NAEP interval, the future ability trend is more optimistic than if using the more recently NAEP interval (2060: 103.17 vs. 101.66 IQ points).

**Table 7:** Projected effect sizes in IQ points based on 1978/80 to 2012 NAEP data (optimistic).

	e_base_	e_minor_	e_pop_	e_p&m_	e_total_
2015	0.14	0.11	-0.09	<0.01	0.17
2020	0.37	0.30	-0.22	0.03	0.48
2025	0.60	0.48	-0.35	0.07	0.81
2030	0.84	0.67	-0.49	0.14	1.16
2035	1.07	0.86	-0.63	0.23	1.53
2040	1.30	1.04	-0.78	0.35	1.91
2045	1.53	1.23	-0.93	0.49	2.32
2050	1.77	1.41	-1.08	0.65	2.75
2055	2.00	1.60	-1.23	0.83	3.20
2060	2.23	1.79	-1.36	1.03	3.68
per decade	0.46	0.37	-0.28	0.21	0.77

Effects for population, based on longer period (more optimistic model). e_base_: change based on White’s ability rise; e_minor_: change based on minorities’ catch up; e_pop_: change based on change of group proportions in population; e_p&m_: change based on interaction of population change and minority catch up; e_total_: sum of all effects.

**Table 8:** Projected effect sizes in IQ points based on 1992 to 2012 NAEP data (pessimistic).

	e_base_	e_minor_	e_pop_	e_p&m_	e_total_
2015	0.02	0.13	-0.09	<0.01	0.06
2020	0.05	0.34	-0.22	0.03	0.20
2025	0.07	0.55	-0.35	0.09	0.36
2030	0.10	0.76	-0.49	0.17	0.54
2035	0.13	0.97	-0.63	0.28	0.75
2040	0.16	1.19	-0.78	0.42	0.98
2045	0.19	1.40	-0.93	0.58	1.24
2050	0.21	1.61	-1.08	0.78	1.52
2055	0.24	1.82	-1.23	0.99	1.83
2060	0.27	2.03	-1.36	1.22	2.16
per decade	0.06	0.42	-0.28	0.25	0.45

Effects for population, based on more recent period (more pessimistic model). e_base_: change based on White’s ability rise; e_minor_: change based on minorities’ catch up; e_pop_: change based on change of group proportions in population; e_p&m_: change based on interaction of population change and minority catch up; e_total_: sum of all effects.

**Fig 2:**
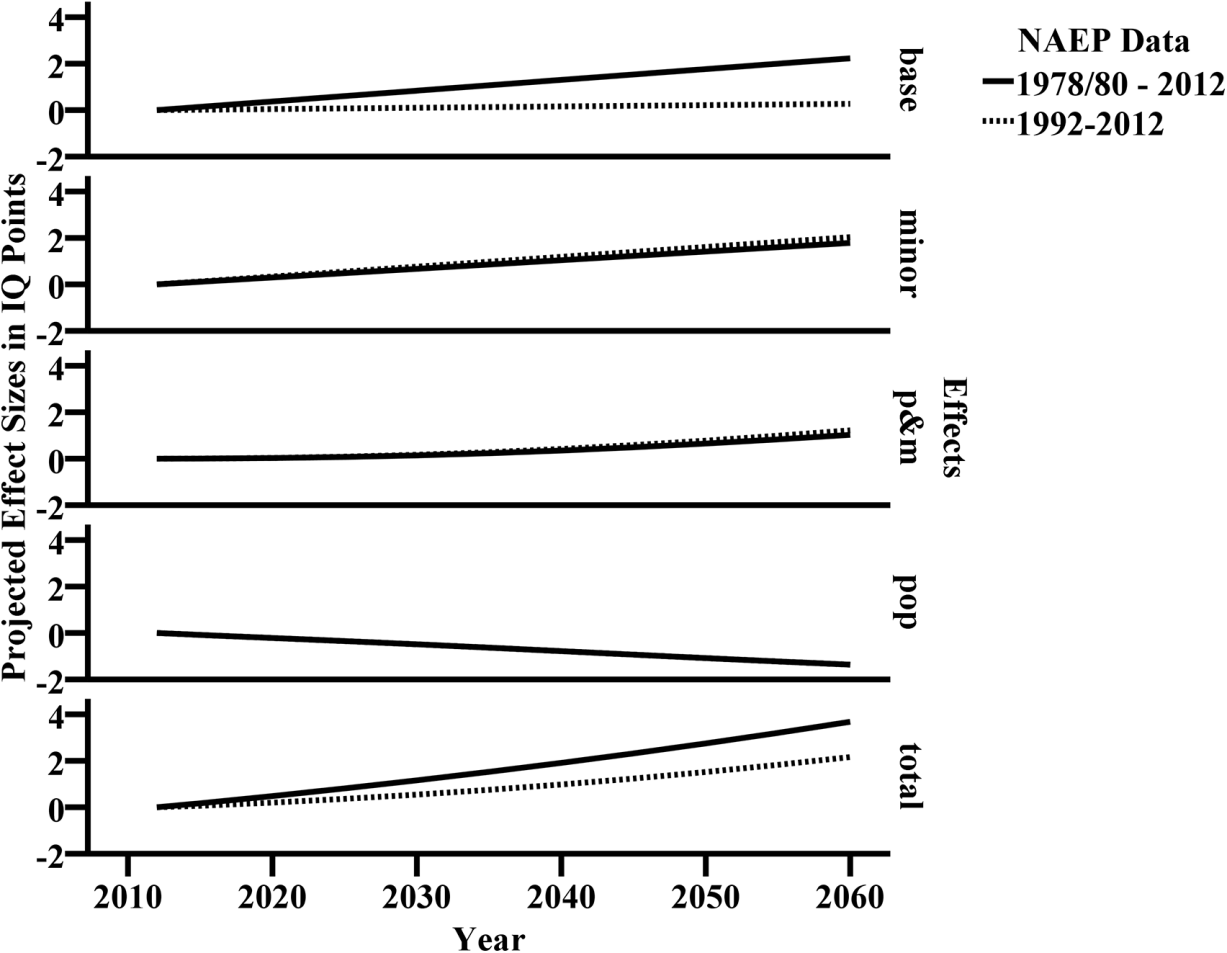
Effect size development from 2020 to 2060. Minority catch up and the interaction between population change and minority catch up are nearly identical for the two models; population change is identical for both models.

To enable better comparisons between the effects of both models, we plotted all effects in Fig 2.

### 2.4. Comparison with worst case and best case models

Tables 9 and 10 show the development of the projected IQ scores in comparison to our adjusted worst and best case models. Each time we used the slopes of the Asian group’s cognitive abilities for the best case model and the slopes of the White group’s cognitive abilities for the worst case model.

**Table 9:** Projected population IQ scores for 2015 to 2060 as well as best and worst case IQ projections based on 1978/80 to 2012 NAEP scores (optimistic).

	Standard	Best Case	Worst Case
2012	99.50	99.50	99.50
2015	99.66	99.91	99.55
2020	99.98	100.61	99.65
2025	100.31	101.31	99.75
2030	100.65	102.01	99.84
2035	101.02	102.70	99.93
2040	101.41	103.38	100.01
2045	101.81	104.07	100.10
2050	102.24	104.75	100.18
2055	102.70	105.44	100.27
2060	103.17	106.14	100.36
Total increase	3.68	6.64	0.87

Cognitive ability projection for population, based on less recent period (more optimistic model). Standard: Projection based on the different cognitive ability increase per year of each race-ethnic group; Best Case: Projection based on best cognitive ability increase per year of all four groups (taken Asians’ development); Worst Case: Projection based on worst cognitive ability increase per year of all four groups (taken Whites’ development).

**Table 10:** Projected population IQ scores for 2015 to 2060 as well as best and worst case IQ projections based on 1992 to 2012 NAEP scores (pessimistic).

	Standard	Best Case	Worst Case
2012	99.50	99.50	99.50
2015	99.56	100.01	99.43
2020	99.70	100.88	99.32
2025	99.86	101.74	99.22
2030	100.04	102.60	99.11
2035	100.25	103.45	98.99
2040	100.48	104.30	98.87
2045	100.73	105.15	98.75
2050	101.01	105.99	98.63
2055	101.32	106.85	98.51
2060	101.66	107.71	98.40
Total increase	2.16	8.21	-1.09

Cognitive ability projection for population, based on more recent period (more pessimistic model). Standard: Projection based on the different cognitive ability increase per year of each race-ethnic group; Best Case: Projection based on best cognitive ability increase per year of all four groups (taken Asians’ development); Worst Case: Projection based on worst cognitive ability increase per year of all four groups (taken Whites’ development).

Both best case models show much higher increases in cognitive abilities in 2060 than our unadjusted projections (3.68 vs. 6.64 IQ points and 2.16 vs. 8.21 IQ points). In comparison, the differences between both worst case models and our unadjusted projections are smaller in 2060 (3.68 vs. 0.87 and 2.16 vs. -1.09).

### 2.5. Economic Estimates

We calculated expectable wealth productivity effects (in US Dollar of 2010/2011). Internationally, one IQ point corresponds to $810 higher average productivity per capita and year. Between 2012 and 2060 it is expected that 17 year olds’ ability level will increase by 2.16 (pessimistic model) or 3.68 IQ (optimistic model), representing a productivity gain of $1,750 to $2,981 (at constant prices). The general FLynn effect (White’s slow ability rise) contributes at about $219 to $1,806, minorities’ catch up at $1,644 to $1,450, demographic change at–$1,102, interaction between minorities’ catch up and demographic change at $988 to $834 US Dollar.

## Discussion

Using two different past intervals and population estimates as basis, we come to a at first sight rather optimistic prediction of future cognitive development in the US: Ability levels will, on average, rise between two and four IQ points. There is no prediction of a decline or disaster. Because cognitive ability contributes to economic productivity, positive prediction at a plus of around $2,000 to $3,000 US Dollar per capita and year for productivity is possible.

However, compared with the 20^th^ century internationally usual ability rise of about 2.83 IQ per decade [4] this development for a 48 year interval is rather disappointing. The average rise per decade is only *dec* = 0.76 or *dec* = 0.45 IQ points, roughly a quarter or sixth of the past international development. Also compared to the somewhat smaller US and British rise of *dec* = 2.31 [5], the predicted US rise until 2060 is rather small (a third to a fifth). This increase is also smaller than the past NAEP-based rise of *dec* = 1.17 IQ points. Minimum and maximum estimations based on Whites’ and Asians’ past developments may give the frame for what is possible (from *dec* = -0.23 to *dec* = 1.71).

What could be the reasons for this rather tiny expected increase? (1) We use results from the oldest age group, the 17-year old group. They give the best source for the ability level of adults, the largest and for society including economy most important age group. But FLynn effect research has shown that there is an “age-FLynn-decline” [9, 44]. It seems to be that past improvements in environmental conditions from nutrition to family and pre-school education mainly speeded up child development. (2) NAEP is more a measure of crystallized than of fluid intelligence. Past rises in crystallized intelligence were smaller than in fluid intelligence [44, 45]. The causes for this difference are not finally found, knowledge has less increased, figural reasoning more (this is no explanation). One reason might be that because of height and brain size increases, more physiological factors as nutrition and health have caused the secular rise in IQ. These factors are more relevant for fluid intelligence. (3) There are limits to cognitively relevant environmental improvability. Environmental improvements reach a point of diminishing returns. (4) Past development may have approached the neurobiological limits of modifiability. However, it is unknown where these limits are. (5) Environments and cultures may have deteriorated in important aspects as cognitively stimulating subjects are replaced by less stimulating ones (e.g. mathematics and language by sports, presentation techniques or cultural awareness), more graduates from the lower end of the academic proficiency distribution of a cohort become teachers [46], replacement of meritoric orientations (e.g. the use of central and objective exams for decisions) by procedures selectively benefiting favored groups [47]. (6) And, as the tables show, demographic change has a lowering effect of about -0.28 IQ per decade. However, the negative effect is only about a tenth of the standard positive effect of *dec* = 2 to 3 IQ points and can explain only a small part of the reduction of the FLynn effect.

The GDP effects were very roughly estimated not considering generally continuing growth. The numbers of $2,000 to $3,000 US Dollar stand only for the surplus due to increased cognitive ability neglecting long-term cumulative effects. Other models [17, 48] considered such baseline growth and the effects of cognitive ability on it. Hanushek et al. see the US GDP in 2060 at around $90,000 US Dollar (in constant 2012 dollars) [17], Meisenberg at $81,000 US Dollar [48]. However, all these positive GDP predictions are probably overestimated: The generally increasing complexity level of production and innovation will raise the bar even for the preservation of the past achieved productivity level not to mention its progress. The internal dynamics of cognitive improvement as well as of technological and organizational modernization may reach a manageable limit.

A second problem deals with possible tipping points in the upward trend of cognitive ability. There is evidence for the end or even reverse of the FLynn effect in some countries as Norway [49], Denmark [50], England [51] and Finland [52]. The interpretation is that immigration of lower cognitive ability groups (apart from initial language problems), lower fertility rates, and higher generation length among the well educated as well as limits of environmental improvability have stopped the upward trend and will lead in future to cognitive declines. Such developments may be boosted by anti-meritoric “reforms” as the substitution of ability based admission standards by political criteria (an example in this direction for New York is given by Borland [53]) or affirmative action and affirmative grading [47, 54–56]. Such approaches to a problem may become more probable with increasing heterogeneity and ability heterogeneity following ethnical groupings.

However, there can come also some important improvements. Less in school education but more in the field of health: Prenatal care, neuroenhancement and therapies against cognitive decline in later adulthood can have substantial positive effects on individuals’ and societies’ competence levels [57]. Health also includes breast-feeding [58]. Further environmental interventions with today known effects include pre-school education (potential in developing nations and for immigrants) and reading to the child by parents and kindergarten teachers [59]. Cognitive training can be added [60–61]. A combination of today known but in future more applied practices and with today unknown and in the next decades developed new health focused interventions can have substantial effects. Finally, the predictions also reveal an increasing ability homogeneity among Whites, Blacks and Hispanics. This may strengthen meritoric principles in education and the working world supporting cognitive development at the level of nations.

## Appendix: Derivation of the equations of the four postulated effects and total effect (e_total_)

$$\begin{array}{l} {\bar{IQ}}_{y,tp}=\sum_{r}\;p_{y,r}\;*\;{\bar{IQ}}_{y,tp,r}\\ \;=\sum_{r}\;p_{y,r}\;*\;({\bar{IQ}}_{2012,tp,r}+m_{tp,r}\;*\;(y-2012))\\ \;=\sum_{r}\;(p_{y,r}\;*\;{\bar{IQ}}_{2012,tp,r}+p_{y,r}\;*\;m_{tp,r}\;*\;(y-2012))\\ \;=\sum_{r}\;p_{y,r}\;*\;{\bar{IQ}}_{2012,tp,r}+(y-2012)\;*\;\sum_{r}\;p_{y,r}\;*\;m_{tp,r}\\ \;=\sum_{r}\;(p_{2012,r}+(p_{y,r}-p_{2012,r}))\;*\;{\bar{IQ}}_{2012,tp,r}+(y-2012)\;*\;\sum_{r}\;p_{y,r}\;*\;m_{tp,r}\\ \;=\sum_{r}\;(p_{2012,r}\;*\;{\bar{IQ}}_{2012,tp,r}+(p_{y,r}-p_{2012,r})\;*\;{\bar{IQ}}_{2012,tp,r})+(y-2012)\;*\;\sum_{r}\;p_{y,r}\;*m_{tp,r}\\ \;=\sum_{r}\;p_{2012,r}\;*\;{\bar{IQ}}_{2012,tp,r}+\sum_{r}\;(p_{y,r}-p_{2012,r})\;*\;{\bar{IQ}}_{2012,tp,r}+(y-2012)\;*\;\sum_{r}\;p_{y,r}\;*m_{tp,r}\\ \;={\bar{IQ}}_{2012,tp}+\sum_{r}\;(p_{y,r}-p_{2012,r})\;*\;{\bar{IQ}}_{2012,tp,r}+(y-2012)\;*\;\sum_{r}\;p_{y,r}\;*m_{tp,r} \end{array}$$

$$\begin{array}{l} e_{total;y,tp}={\bar{IQ}}_{y,tp}-{\bar{IQ}}_{2012,tp}\\ \;=\sum_{r}(p_{y,r}-p_{2012,r})\;*\;{\bar{IQ}}_{2012,tp,r}+(y-2012)\;*\;\sum_{r}p_{y,r}\;*\;m_{tp,r}\\ \;=e_{pop;y,tp}+(y-2012)\;*\;\sum_{r}p_{y,r}\;*\;m_{tp,r}\\ \;=e_{pop;y,tp}+(y-2012)\;*\;\sum_{r}p_{y,r}\;*\;(m_{tp,white}+(m_{tp,r}-m_{tp,white}))\\ \;=e_{pop;y,tp}+(y-2012)\;*\;(\sum_{r}p_{y,r}\;*\;m_{tp,white}+\sum_{r}p_{y,r}\;*\;(m_{tp,r}-m_{tp,white}))\\ \;=e_{pop;y,tp}+(y-2012)\;*\;(m_{tp,white}+\sum_{r}p_{y,r}\;*\;(m_{tp,r}-m_{tp,white}))\\ \;=e_{pop;y,tp}+e_{base;y,tp}+(y-2012)\;*\;\sum_{r}p_{y,r}\;*\;(m_{tp,r}-m_{tp,white})\\ \;=e_{pop;y,tp}+e_{base;y,tp}+(y-2012)\;*\;\sum_{r}(p_{2012,r}+(p_{y,r}-p_{2012,r}))\;*\;(m_{tp,r}-m_{tp,white})\\ \;=e_{pop;y,tp}+e_{base;y,tp}+e_{minor;y,tp}+(y-2012)\;*\;\sum_{r}(p_{y,r}-p_{2012,r})\;*\;(m_{tp,r}-m_{tp,white})\\ \;=e_{pop;y,tp}+e_{base;y,tp}+e_{minor;y,tp}+e_{p\&m;y,tp} \end{array}$$

## References

[1] Flynn JR. The mean IQ of Americans: Massive gains 1932 to 1978. Psychol Bull. 1984;95: 29–51.

[2] Rundquist EA. Intelligence test scores and school marks of high school seniors in 1929 and 1933. Sch Soc. 1936;43: 301–304.

[3] Lynn R. IQ in Japan and the United States shows a growing disparity. Nature. 1982;297: 222–223.

[4] Pietschnig J, Voracek M. One century of global IQ gains: A formal meta-analysis of the Flynn effect (1909–2013). Perspect Psychol Sci. 2015;10: 282–306. 10.1177/1745691615577701 25987509

[5] Trahan LH, Stuebing KK, Fletcher JM, Hiscock M. The Flynn effect: A meta-analysis. Psychological Bulletin. Psychol Bull. 2014;140: 1332–1360. 10.1037/a0037173 24979188 PMC4152423

[6] Meisenberg G, Woodley MA. Are cognitive differences between countries diminishing? Evidence from TIMSS and PISA. Intelligence. 2013;41: 808–816.

[7] Rindermann H. African cognitive ability: Research, results, divergences and recommendations. Pers Individ Dif. 2013;55: 229–233.

[8] Rindermann H, Schott T, Baumeister AEE. FLynn effect in Turkey. Intelligence. 2013;41:178–180.

[9] Rindermann H, Thompson J. Ability rise in NAEP and narrowing ethnic gaps?. Intelligence. 2013;41: 821–831.

[10] Autor DH, Price B. The changing task composition of the US labor market: An update of Autor, Levy, and Murnane (2003). Cambridge: MIT. 2013: unpublished manuscript.

[11] Dobbs R, Madgavkar A, Barton D, Labaye E, Manyika J, Roxburgh C, et al The world at work: Jobs, pay, and skills for 3.5 billion people. New York: McKinsey; 2012.

[12] Hunt E. Will we be smart enough? A cognitive analysis of the coming workforce. New York: Russell Sage Foundation; 1995.

[13] Bishop JH. Is the test score decline responsible for the productivity growth decline? Am Econ Rev. 1989;79: 178–197.

[14] Hunt E, Madhyastha TM. Cognitive demands of the workplace. J Neurosci Psychol Econ. 2012;5: 18–37.

[15] Schmidt FL, Hunter JE. General mental ability in the world of work: Occupational attainment and job performance. J Pers Soc Psychol. 2004;86: 162–173. 14717634 10.1037/0022-3514.86.1.162

[16] Wai J. Investigating America’s elite: Cognitive ability, education, and sex differences. Intelligence. 2013;41: 203–211.

[17] Hanushek EA, Peterson PE, Woessmann L. Endangering prosperity Washington: Brookings; 2013.

[18] Summers L. Foreword In: Hanushek EA, Peterson PE, Woessmann L, editors. Endangering prosperity. Washington: Brookings; 2013 pp. VII–IX.

[19] Heckman JJ, LaFontaine PA (2007) The American high school graduation rate: Trends and levels. IZA Discussion Paper. 2017:3216; 1–62. 10.1162/rest.2010.12366 PMC2900934 20625528

[20] Wadhwa V. Why America is losing the global race to capture entrepreneurial talent Philadelphia: Wharton; 2012.

[21] Kirsch I, Braun H, Yamamoto K. America’s perfect storm: Three forces changing our nation’s future Princeton: Educational Testing Service (ETS); 2007.

[22] Woodley MA, te Nijenhuis J, Murphy R. Were the Victorians cleverer than us? The decline in general intelligence estimated from a meta-analysis of the slowing of simple reaction time. Intelligence. 2013;41: 843–850.

[23] Nyborg H. The decay of Western civilization: Double relaxed Darwinian selection. Pers Individ Dif. 2012;53: 118–125.

[24] Dronkers J, van der Velden R, Dunne A. Why are migrant students better off in certain types of educational systems or schools than in others? Eur Educ Res J. 2012;11: 11–44.

[25] OECD. PISA 2009 results (Volume II). Paris: OECD; 2010.

[26] Rindermann H, Thompson J. The cognitive competences of immigrant and native students across the world: An analysis of gaps, possible causes and impact. J Biosoc Sci. 2014 11 7 10.1017/S0021932014000480 25376963

[27] te Nijenhuis J, de Jong MJ, Evers A, van der Flier H. Are cognitive differences between immigrant and majority groups diminishing? Eur J Pers. 2004;18: 405–434.

[28] Passel JS, Cohn DV. US population projections: 2005–2050 Washington: Pew Research Center; 2008.

[29] Neal D. Why has black-white skill convergence stopped? In: Hanushek EA & Welch F, editors. Handbook of the economics of education (Volume I). Amsterdam: North-Holland; 2006 pp. 511–576.

[30] Barton PE, Coley RJ. The Black-White achievement gap: When progress stopped Princeton: Education Testing Service; 2010 Available: http://www.ets.org/Media/Research/pdf/PICBWGAP.pdf. Accessed 2011 Oct 14.

[31] Rushton JP, Jensen AR. Thirty years of research on Black-White differences in cognitive ability. Psychol Public Policy Law. 2005;11: 235–294.

[32] National Center for Education Statistics. Trends in academic progress 2012 Washington: US Department of Education, NCES 2013–456; 2013 Available: http://nces.ed.gov/nationsreportcard/subject/publications/main2012/pdf/2013456.pdf. Accessed 2014 Jan 10.

[33] Rindermann H, Baumeister AEE. Validating the interpretations of PISA and TIMSS tasks. Int J Test. 2015;15: 1–22.

[34] Kaufman SB, Reynolds MR, Liu X, Kaufman AS, McGrew KS. Are cognitive g and academic achievement g one and the same g?. Intelligence. 2012;40: 123–138.

[35] Deary IJ, Strand S, Smith P, Fernandes C. Intelligence and educational achievement. Intelligence. 2007;35: 13–21.

[36] Frey MC, Detterman DK. Scholastic assessment or g?. Psychol Sci. 2004;15: 373–378. 15147489 10.1111/j.0956-7976.2004.00687.x

[37] Meisenberg G, Lynn R. Intelligence: A measure of human capital in nations. J Soc Polit Stud. 2011;36: 421–454.

[38] Rindermann H, Michou CD, Thompson J. Children’s writing ability: Effects of parent’s education, mental speed and intelligence. Learn Individ Differ. 2011;21: 562–568.

[39] National Center for Education Statistics. NAEP data explorer; 2013. Available: http://nces.ed.gov/nationsreportcard/lttdata. Accessed 2013 Sept 16.

[40] US Census Bureau, Population. Projections of the population by sex, race, and Hispanic origin for the United States: 2015 to 2060 (NP2012-T4). Dec 2012. Available: http://www.census.gov/population/projections/data/national/2012/summarytables.html. Accessed 2013 Oct 22.

[41] US Census Bureau, Population Division. Annual Estimates of the Resident Population by Sex, Race, and Hispanic Origin for the United States, States, and Counties: April 1, 2010 to July 1, 2012. Jun 2013. Available: www.census.gov/popest/data/historical/2010s/vintage_2012/national.html. Accessed 2013 Oct 22.

[42] Lynn R, Vanhanen T. Intelligence A unifying construct for the social sciences. London: Ulster Institute for Social Research; 2012.

[43] US Census Bureau. The Asian Population: 2010. Washington: US Department of Commerce. Mar 2012. Available: www.census.gov/prod/cen2010/briefs/c2010br-11.pdf. Accessed 2014 Nov 10.

[44] Lynn R. Who discovered the Flynn effect? A review of early studies of the secular increase of intelligence. Intelligence. 2013;41: 765–769.

[45] Flynn JR. Are we getting smarter? Rising IQ in the twenty-first century. Cambridge: Cambridge University Press; 2012.

[46] Eide E, Goldhaber D, Brewer D. The teacher labour market and teacher quality. Oxford Rev Econ Policy. 2004;20: 230–244.

[47] Sowell T. Inside American education: The decline, the deception, the dogmas New York: Free Press; 1993.

[48] Meisenberg G. Cognitive human capital and economic growth in the 21st century In: Abrahams T, editor. Economic growth in the 21st century. New York: Nova Publishers; 2014 pp. 49–106.

[49] Sundet JM, Barlaug DG, Torjussen TM. The end of the Flynn effect? A study of secular trends in mean intelligence test scores of Norwegian conscripts during half a century. Intelligence. 2004;32: 349–362.

[50] Teasdale TW, Owen DR. Secular declines in cognitive test scores: A reversal of the Flynn Effect. Intelligence. 2008;36: 121–126.

[51] Shayer M, Ginsburg D. Thirty years on–a large anti-Flynn effect (II)? 13- & 14-year-olds. Piagetian tests of formal operations norms 1976-2006/7. Br J Educ Psychol. 2009;79: 409–418. 10.1348/978185408X383123 19055872

[52] Dutton E, Lynn R . A negative Flynn effect in Finland, 1997–2009. Intelligence. 2013;41: 817–820.

[53] Borland JH. New York city gets it wrong again. The Creativity Post. 14 Sep 2012. Available: http://www.creativitypost.com/education/new_york_city_gets_it_wrong_again. Accessed 2013 Oct 12.

[54] Harber KD. Feedback to minorities: Evidence of a positive bias. J Pers Soc Psychol. 1998;74: 622–628. 9523409 10.1037//0022-3514.74.3.622

[55] Sowell T. Affirmative action around the world: An empirical study. New Haven: Yale University Press; 2004.

[56] Farron S. The affirmative action hoax. Quezon City: New Century Books; 2010.

[57] Stough C, Camfield D, Kure C, Tarasuik J, Downey L, Lloyd J, et al Improving general intelligence with a nutrient-based pharmacological intervention. Intelligence. 2011;39: 100–107.

[58] Protzko J, Aronson J, Blair C. How to make a young child smarter. Perspect Psychol Sci. 2013;8: 25–40. 10.1177/1745691612462585 26172250

[59] Baumeister AEE, Rindermann H, Barnett WS. Crèche attendance and children’s intelligence and behavior development. Learn Individ Differ. 2014;30: 1–10.

[60] Jaeggi SM, Buschkuehl M, Jonides J, Perrig WJ. Improving fluid intelligence with training on working memory. Proc Natl Acad Sci. 2008;105: 6829–6833. 10.1073/pnas.0801268105 18443283 PMC2383929

[61] Rindermann H, Baumeister AEE. Effectiveness of Klauer’s cognitive training with immigrant students, special-needs students and elderly people. Psychologie in Erziehung und Unterricht. 2013;60: 121–132.

